# Folliculocentric eruption following stem cell transplantation

**DOI:** 10.1016/j.jdcr.2024.01.035

**Published:** 2024-03-26

**Authors:** Michael J. Diaz, Mahtab Forouzandeh, Tyler Werbel, Kiran Motaparthi

**Affiliations:** a College of Medicine, University of Florida, Gainesville, Florida; b Department of Dermatology, University of Florida, Gainesville, Florida

**Keywords:** acute myeloid leukemia, bone marrow transplant, busulfan, pediatric, toxic erythema of chemotherapy

## History

**Fig 1:**
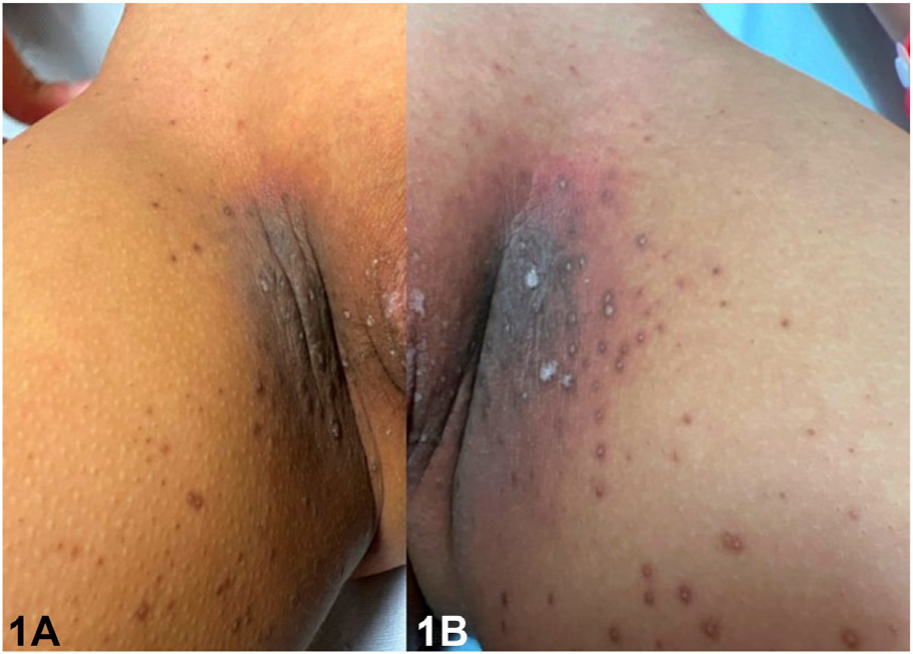

**Fig 2:**
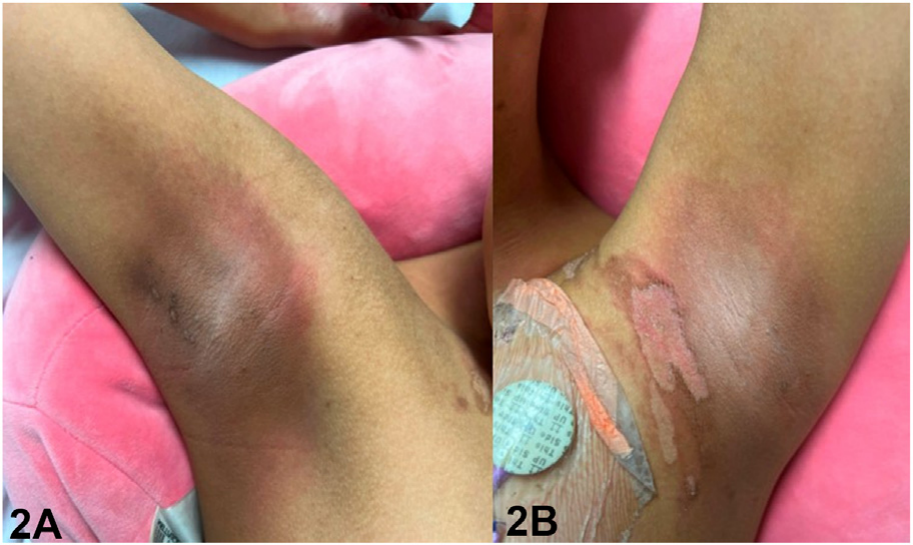

**Fig 3:**
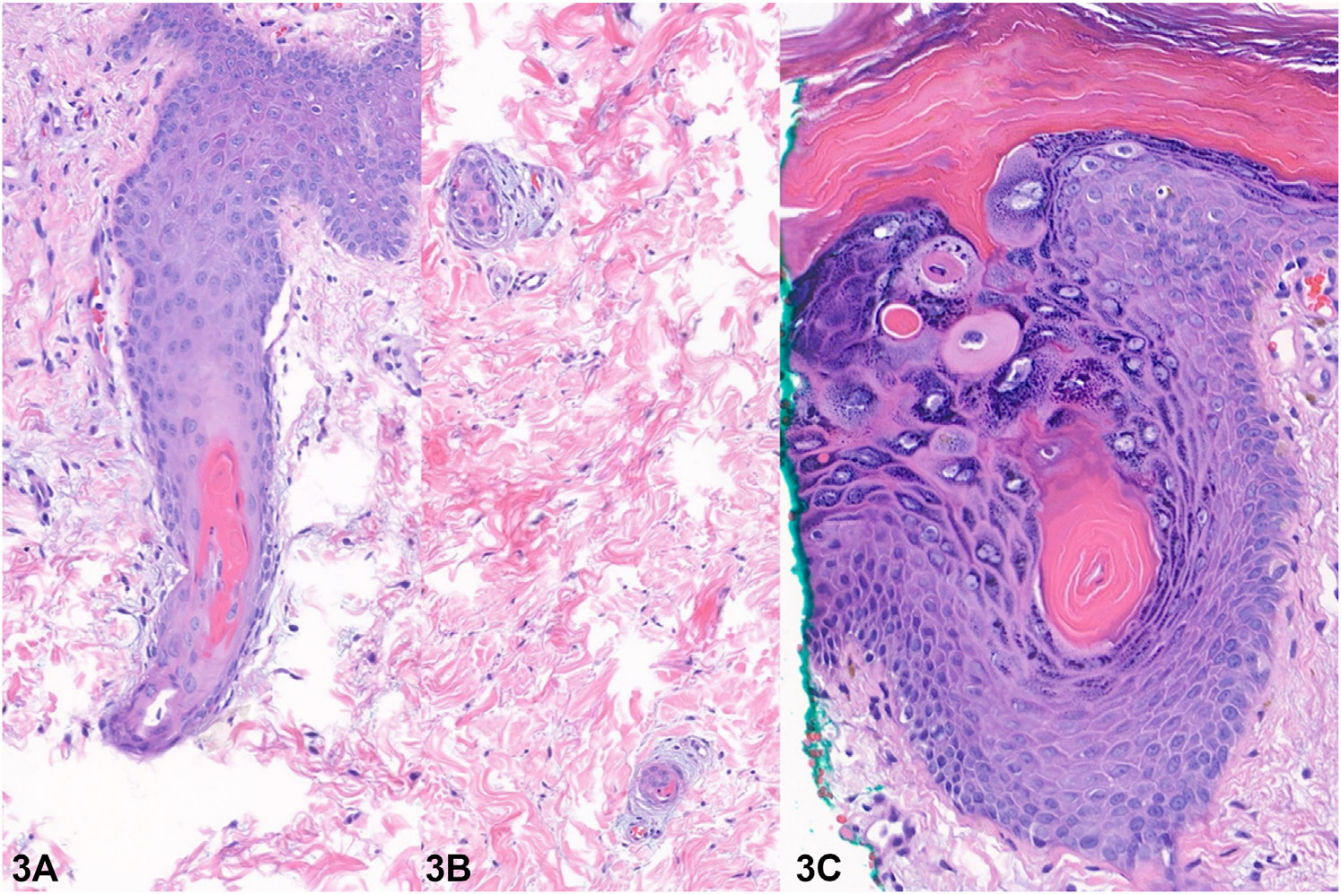

A 12-year-old girl with a history of acute myeloid leukemia presented with a 2-week history of perifollicular hyperpigmented papules. Onset was 44 days following haploidentical stem cell transplant, preceded by conditioning chemotherapy with busulfan and fludarabine. Examination revealed numerous hyperpigmented papules and superficial desquamation involving the bilateral axillae, inguinal folds, and abdominal flexures. Papules distributed on the inguinal area coalesced within the inguinal folds (Fig 1). Erythema and superficial desquamation were also observed in the axillae (Fig 2). Punch biopsy revealed dysmaturation overlying an acanthotic and papillomatous epidermis. Follicular plugging with dyskeratosis, dysmaturation, and syringosquamous metaplasia were also seen (Fig 3).

**Question 1: Which of the following is the most likely diagnosis?**

A. Acute graft-versus-host disease (aGVHD)
B. Lichen planopilaris (LPP)
C. Toxic erythema of chemotherapy (TEC)
D. Candidal intertrigo
E. Toxic epidermal necrolysis (TEN)

**Answers:**

A. aGVHD – Incorrect. The absence of an exanthematous to necrolytic clinical presentation in addition to the histopathological findings (dyskeratosis, follicular plugging, and syringosquamous metaplasia) are inconsistent with aGVHD.
B. LPP – Incorrect. LPP is a scarring alopecia characterized by perifollicular erythema and scaling. While LPP demonstrates follicular distribution, this patient also presented with desquamation of the inguinal folds and axillae.
C. TEC – Correct. TEC encompasses a spectrum of cutaneous eruptions triggered by chemotherapeutic drugs.^1^ Latency is variable, but findings classically appear within 2 days to 3 weeks of chemotherapy initiation and are self-limited, resolving with desquamation followed by postinflammatory hyperpigmentation.^2^ The perifollicular distribution, together with intertriginous desquamative erythema, recent chemotherapy, and consistent pathologic findings support a diagnosis of TEC secondary to the busulfan conditioning regimen. Similar presentations have been previously reported with use of busulfan-containing regimens.^3^
D. Candidal intertrigo – Incorrect. Intertrigo complicated by candidiasis is characterized by a flexural eruption with erythema, maceration, and peripheral scale with pustules; satellite lesions are variable. Additionally, no fungal elements were identified on histopathology.
E. TEN – Incorrect. The mild clinical presentation, lack of mucosal involvement, latency of more than 1 month, and evidence of dysmaturation on histopathology are not consistent with TEN.

**Question 2: In differentiating TEC from aGVHD following stem cell transplant, which of the following clinical features is more likely to be observed in TEC?**

A. Morbilliform exanthem
B. Severe mucositis
C. Diarrhea
D. Hepatitis
E. Flexural distribution

**Answers:**

A. Morbilliform exanthem – Incorrect. aGVHD typically presents as a morbilliform exanthem or in severe cases with epidermal necrolysis.
B. Severe mucositis – Incorrect. Severe mucositis is more typical of TEN and can also be observed in aGVHD. Even in severe cases of TEC, mucositis is typically mild.
C. Diarrhea – Incorrect. Systemic involvement including gastrointestinal disease and hepatitis is more characteristic of aGVHD.
D. Hepatitis – Incorrect. Systemic involvement including hepatitis is more characteristic of aGVHD.
E. Flexural distribution – Correct. TEC most commonly affects intertriginous areas or skin folds of flexural regions such as the axillae and groin.

**Question 3: Which of the following statements provides the appropriate management of this condition?**

A. Methylprednisone
B. Topical emollients or corticosteroids
C. Hydroxychloroquine
D. Fluconazole
E. Intravenous immunoglobulin

**Answers:**

A. Methylprednisone – Incorrect. High-dose systemic corticosteroids can be used for the treatment of aGVHD.
B. Topical emollients or corticosteroids – Correct. TEC is most commonly managed with supportive care, including topic emollients and mild to high-potency topical corticosteroids.^4^^,^^5^
C. Hydroxychloroquine – Incorrect. Hydroxychloroquine may be administered for LPP.
D. Fluconazole – Incorrect. Fluconazole is the systemic treatment of choice for candidal intertrigo.
E. Intravenous immunoglobulin – Incorrect. In combination with systemic corticosteroids, intravenous immunoglobulin is utilized in the treatment of TEN.

## Conflicts of interest

None disclosed.
